# Popliteal Cysts in Paediatric Patients: Clinical Characteristics and Imaging Features on Ultrasound and MRI

**DOI:** 10.1155/2011/751593

**Published:** 2011-05-05

**Authors:** Henning Neubauer, Henner Morbach, Tobias Schwarz, Clemens Wirth, Hermann Girschick, Meinrad Beer

**Affiliations:** ^1^Department of Paediatric Radiology, Institute of Radiology, University Hospital Wuerzburg, 97080 Wuerzburg, Germany; ^2^Department of Paediatrics, University Hospital Wuerzburg, 97080 Wuerzburg, Germany

## Abstract

Popliteal cysts, or Baker cysts, are considered rare in children and may exhibit particular features, as compared with adults. We studied data from 80 paediatric patients with 55 Baker cysts, examined over a period of 7 years, and correlated clinical presentation with findings on ultrasonography and MRI. Prevalence of popliteal cysts was 57% in arthritic knees, 58% with hypermobility syndrome, and 28% without risk factors. Only one patient had a trauma history and showed an ipsilateral cyst. Mean cyst volume was 3.4 mL; cysts were larger in boys. Patients with arthritis had echogenic cysts in 53%. Cyst communication with the joint space was seen in 64% on ultrasonography and 86% on MRI. In conclusion, Baker cysts are a common finding in a clinically preselected paediatric population. Children with Baker cysts should be assessed for underlying arthritis and inherited joint hypermobility, while sporadic Baker cysts appear to be common, as well.

## 1. Introduction

Popliteal cysts or Baker cysts represent a distended gastrocnemio-semimembranosus bursa. First description of popliteal cysts is attributed to Adams in 1840, while Baker studied these cysts in the context of intra-articular pathologies and effusion of the knee joint [1]. Baker cysts are a common finding in adult imaging studies with a reported prevalence ranging from 10% to 41% [2] and are commonly considered an epiphenomenon of underlying inflammatory or degenerative arthropathy in this age group. In contrast, patients from paediatric populations rarely exhibit Baker cysts. Reported prevalence ranges from 2.4% in a small prospective asymptomatic screening population [3] to 6.3% in an MRI study on children with knee pain [4]. Baker cysts appear to be common in juvenile rheumatoid arthritis [5], where studies found popliteal cysts in up to 61% and demonstrated an association with joint effusion [6]. Controversy still prevails on the question whether Baker cysts in children communicate with the internal joint space [7, 8]. Synovial cysts can also be found in locations other than the knee joint [9].

We reviewed data from a paediatric population with clinically suspected Baker cysts in order to evaluate the prevalence and the characteristics of popliteal cysts in clinically defined subgroups on ultrasonography and magnetic resonance imaging. In addition, we studied the presence of cyst communication with knee joint space and the association with joint effusion.

## 2. Patients and Methods

Our study is based on the retrospective data analysis of 80 consecutive patients, who were examined for clinically suspected popliteal cysts at our department between May 2003 and September 2010. Patients were examined and referred by trained paediatricians from the outpatient clinic and the department of paediatric rheumatology at our institution. The study group comprised 34 females and 46 males with a mean age of 8.6 ± 4.7 years (range 1.9*⋯*2 years). Mean age did not show a significant gender difference (males 7.7 ± 3.9 years versus females 9.7 ± 5.5 years; independent sample *t*-test *P* > .05).

All patients underwent routine ultrasonography of the knee for joint pain and/or popliteal swelling. Ultrasonographic examinations (7–14 MHz linear transducers, models Elegra, Sequoia, Acuson 2000, Siemens Medical, Erlangen, Germany) were performed by residents and supervised by expert paediatric radiologists. Both knees were examined in 67 patients, the right knee only in 6, and the left knee only in 7 patients, totalling 147 joint examinations. All joints were scanned in B-mode at the anterior and posterior aspect in transverse and sagittal planes. Popliteal cysts were defined as a well-delineated lesions extending from the space between the tendons of the medial head of the gastrocnemius muscle and the semimembranosus tendon [10]. All cysts were classified as anechoic, hypoechoic, or of mixed internal signal. Doppler sonography was performed in all patients for assessment of synovial hyperperfusion. Colour-encoded pulsed-wave Doppler and/or power Doppler were applied in all patients (Doppler frequency ≥5 MHz, pulse repetition frequency between 500 and 1000 Hz, low wall filter, and gain setting just below the noise level), as deemed appropriate by the examiner. Tapering of the distal cyst contour, ill-defined margins, and perilesional fluid accumulation were considered signs of cyst rupture [10]. Information on three-dimensional volume measurements, evidence of cyst rupture or dissection and the presence of cyst communication with the knee joint space, joint effusion, synovial thickening, and synovial hyperperfusion were taken from the ultrasonography reports. 

Ultrasonography was followed by MRI of the knee joint in 16 of 80 patients (20%) for evaluation of arthritic bone and cartilage lesions and for suspected internal knee derangement, examining 24 of 147 knee joints (16%). Nineteen MRI studies were performed on a 1.5 Tesla scanner (MAGNETOM Symphony, Siemens Medical, Erlangen, Germany), while one recent examination was performed on a 3 Tesla scanner (MAGNETOM Trio, Siemens Medical). The MRI study protocol was individually adapted to the clinical question, but it contained both T1w and PDw/T2w sequences with ≤3 mm slice thickness in at least two spatial dimensions. Six patients with suspected or diagnosed arthritis had received intravenous gadolinium-containing contrast agent. Diagnostic criteria for Baker cysts on MRI included a liquid-isointense signal T2w and corresponding low signal in T1w sequences. The presence of inhomogeneous T2w signal, signs of fluid leakage, internal septations, wall thickening, and peripheral contrast enhancement were recorded. In addition, other causes of popliteal swellings, such as lymphadenopathy, vascular dilatation, or tumorous lesions, and signs of internal knee derangement, including meniscopathy, cruciate ligament rupture, and osteochondral lesions were searched for.

For the present study, all images and reports were reviewed and reevaluated by two paediatric radiologists in consensus. All work in this study was conducted in accordance with the Declaration of Helsinki 1964. Informed consent for all the routine imaging studies had been obtained from the parents and, if possible, from the patient.

### 2.1. Statistics

All data are presented as mean ± standard deviation. Between-groups comparison of categorical data was performed with a Chi-square test or with Fisher's exact test, as appropriate. Differences in mean values between study groups were tested for with the independent sample *t*-test or the paired sample *t*-test, as appropriate. Intergroup comparison for mean age and cyst size was done with the Kruskal-Wallis test (Monte Carlo method). Pearson's bivariate correlation analysis was used to study the association between continuous variables. A binary logistic regression model employing stepwise forward variable selection was fitted to search for, and derive estimates of, significant risk factors. All analyses were performed with the SPSS 13.0 software package for Windows (SPSS Inc., Chicago, Ill,USA).

## 3. Results

### 3.1. Patient Characteristics

Seventeen patients were diagnosed with arthritis (juvenile rheumatoid arthritis *n* = 5, Lyme arthritis *n* = 4, nonclassified oligo- or polyarthritis *n* = 5, and unilateral or bilateral gonarthritis *n* = 3). Another patient suffered from hypophosphatasia. Clinical examination revealed joint hyperlaxity with suspected benign joint hypermobility syndrome in six otherwise healthy patients. In another patient, MRI showed bilateral areas of steroid-induced bilateral osteonecrosis in the distal femur and the proximal tibia. Only one patient had a trauma history and reported a distortion of the knee joint four months prior to the examination. In total, 26 (33%) of our 80 patients had some knee-related risk factors for developing popliteal cysts. Knee pain was reported in 56%, and popliteal swelling was seen in 29% of the study group. One patient was suspected as having PFAPA syndrome (periodic fever, aphthous stomatitis, pharyngitis, and adenitis).

### 3.2. Ultrasonography

Data comparing patients with arthritis, joint hypermobility, and others are outlined in Table 1. Ultrasound identified a total of 55 Baker cysts in 147 examined knee joints (37%) among 46 of all 80 patients. Twenty-four (44%) of the 55 cysts were located at the right and 31 (56%) at the left (Chi-square test *P* = .307). Of 46 patients with Baker cysts, 37 had unilateral and 9 had bilateral popliteal cysts. The presence of a Baker cyst on ultrasonography was significantly related to popliteal swelling (Chi-square test *P* < .001), but not to reported knee pain (Chi-square test *P* = .426). The diagnostic criterion of a palpable popliteal swelling had a sensitivity of 65%, a specificity of 92%, a positive predictive value of 84%, a negative predicted value of 82%, and a diagnostic accuracy of 82%, as compared with the sonographic findings as reference. 

**Figure 1:**
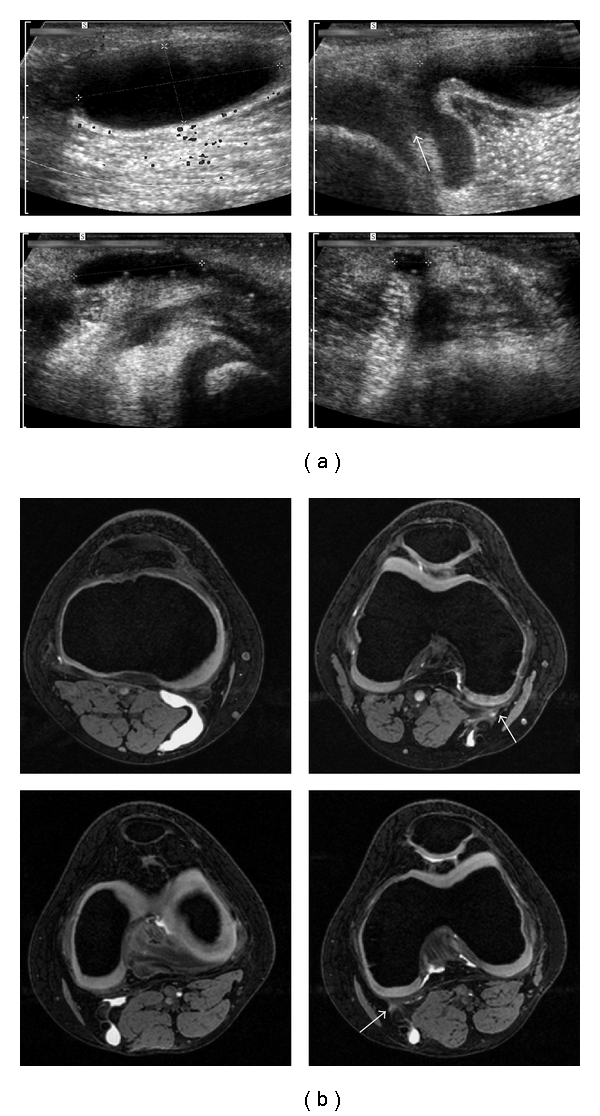
Bilateral Baker cysts in an 11-year-old boy diagnosed with joint hypermobility syndrome. (a) Ultrasonography showed anechoic cysts with a volume of 5.2 mL (right knee, upper row) and 2 mL (left knee, lower row). Communication with the joint space on the right side was described in the ultrasound report, though the documented image of the assumed site of communication (arrow) is not completely conclusive. Patient information blurred. (b) Magnetic resonance imaging in the same patient (3 Tesla MAGNETOM Trio, Siemens, transversal DESS sequence) proved Baker cysts of the right (upper row) and the left (lower row) popliteal fossa in the absence of other pathologies. Both cysts extend towards the joint space, exhibit fluid signal in the vicinity of the articular capsule and were assessed as communicating cysts.

**Figure 2:**
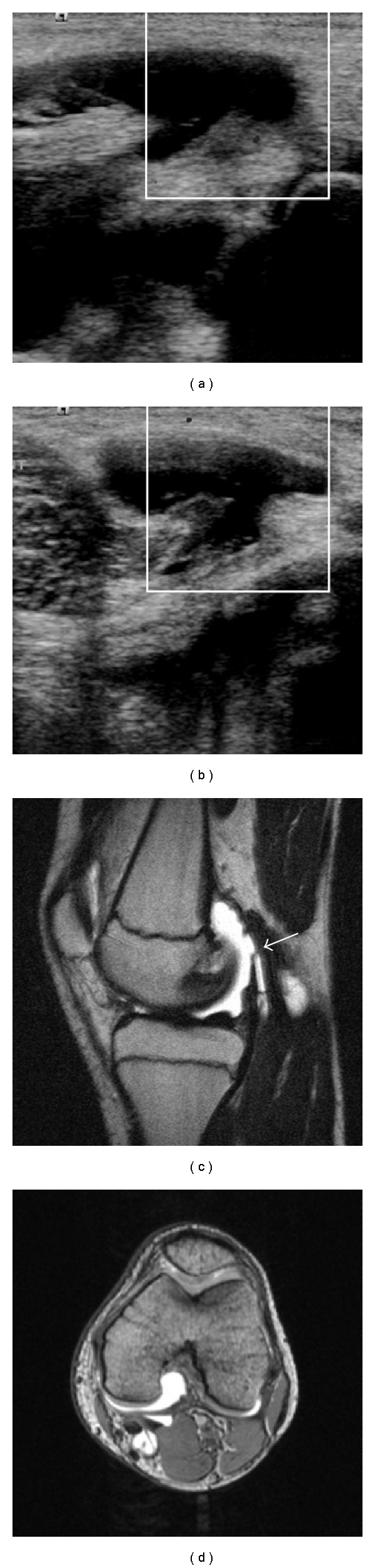
Baker cyst of the left knee in a 14-year-old girl with juvenile rheumatoid arthritis. Ultrasonography demonstrated the cystic lesion with 2.8 mL volume and echogenic sedimentation (a,b). Magnetic resonance imaging (1.5 Tesla MAGNETOM Symphony, Siemens, sagittal proton-density and T2-weigthed imaging, transversal DESS sequence) depicts the cyst with homogenous liquid signal and suggests continuity between cyst and joint space in the presence of joint effusion.

Mean cyst size was 3.4 ± 2.9 mL, ranging from 0.5 mL to 16 mL, with a larger volume in boys (4.4 ± 4 ml versus 2.3 ± 1.5 mL, independent sample *t*-test *P* = .048). There was no correlation between patient age and the size of the popliteal cyst (Pearson's *r* = 0.090, *P* = .512). All cysts showed the characteristic location in the medial popliteal fossa with their largest transversal diameter approximately located at the level of the tibiofemoral joint space. Cyst extension beyond the popliteal fossa was not observed. Sixty-five percent (*n* = 36) of the cysts showed well-delineated, nonthickened cyst wall and anechogenic internal signal (Figure 1), whereas 35% (*n* = 19) had homogenous hypoechoic internal signal or mixed echogenicity (*n* = 15), cyst wall thickening (*n* = 6), septations (*n* = 5), or a combination of these findings (Figure 2). Cysts with extraluminal fluid accumulation indicating rupture or dissection, soft-tissue lesions, or aneurysms of the large popliteal vessels were not seen in our study group. 

Thirty-five cysts (64%) were found to communicate with the posterior knee joint space. These cysts were significantly larger than cysts without communication to the internal joint space (4.4 ± 3.2 mL versus 1.7 ± 1.3 mL, independent sample *t*-test *P* < .001). There was no association between the proportion of communicating cysts and patient age. Effusion of varying degree was seen in 27 (18%) joints. A significant association existed between the presence of joint effusion and the presence of Baker cysts, but not with cyst size, as we found 15 cysts in 27 joints with and 40 cysts among 120 joints without joint effusion (Chi-Square test *P* = .031). Synovial thickening and hyperperfusion were demonstrated in 11 patients. All of these patients were diagnosed with arthritis, and eight of them had Baker cysts, including two cases of bilateral cysts. The presence of synovitis was significantly associated with popliteal cysts (Fisher's exact test *P* = .020). Arthritis patients with Baker cysts were significantly older than patients with non-arthritis-related Baker cysts (11.3 ± 4.5 versus 7.1 ± 4 years, independent sample *t*-test *P* = .001), often female (50% versus 16%, Chi-Square test *P* = .041), and showed a higher frequency of nonanechogenic cysts (53% versus 17%, Chi-Square test *P* = .005) and joint effusion (73% versus 13%, Chi-Square test *P* < .001). Baker cysts in these patients, however, were comparable in size to other patients (*P* > .05). 

On logistic binary regression with per-patient analysis, the presence of Baker cysts was significantly associated with male sex (odds ratio 2.9, 95% confidence interval 1.2*⋯*7.4) and the presence of arthritis or joint hypermobility (odds ratio 3.2, 95% CI 1.1*⋯*9.0).

### 3.3. MR Imaging

We examined 24 knee joints (right side only *n* = 6, left side only *n* = 2, bilateral examination *n* = 8) in 16 patients (Table 1). All 14 knee joints of 11 patients with ultrasonographically identified Baker cyst showed a corresponding cyst on MRI, while MR imaging did not identify any additional cysts or noncystic popliteal pathologies. The mean cyst volume measured on MRI did not significantly differ from the corresponding ultrasonography measurement (4.0 ± 2.8 mL versus 3.6 ± 2.8 mL, paired sample *t*-test *P* = .253). Two cysts showed septations and one cyst exhibited discrete peripheral contrast enhancement. Correlation with the ultrasonographic examination showed wall thickening of the contrast-enhancing cyst on ultrasound, while the septations visible on MRI in two cysts were not depicted on US. Twelve (86%) of 14 Baker cysts were found to communicate with the joint space on MRI. The remaining two cysts were classified as noncommunicating cysts on both US and MRI. Increased internal echogenicity seen in four cysts was not associated with any discernible signal alteration on MRI. Joint effusion was seen both with MRI and US in 5 joints, with MRI only in 2 cases and with US only in 1 case. Absence of joint effusion in both imaging modalities was documented in 16 joints. Our only patient with a history of knee joint distortion showed a linear signal increase in the posterior horn and the pars intermedia of the lateral meniscus without extension to the meniscal surface indicating degenerative meniscopathy.

## 4. Discussion

Our study group is so far the largest reported series of consecutive paediatric patients with popliteal cysts. One important finding is that patient referral by paediatricians trained in rheumatology resulted in a high rate of positive findings in this study cohort. Clinical examination for popliteal swelling, therefore, can effectively focus imaging studies performed in patients with suspected popliteal cysts. In contrast, a large MRI study with 393 participants examined for knee pain, popliteal swelling, or both, over the course of six years, only found 25 cysts with a prevalence of 6% [4]. From our experience, knee pain alone is a very unspecific predictor of popliteal cysts and should be supported with other clinical findings.

The high prevalence of unilateral and bilateral Baker cysts in arthritis patients from our study compares well to the data presented by Szer et al. [6], who found cysts in 61% of patients with juvenile rheumatoid arthritis. Other studies showed a lower prevalence in paediatric arthritis patients of less than 1%, 10%, and 14% [11–13]. We assume that this variation is most likely the result of a selection bias on behalf of the referring clinicians. As in [6], we showed a relation between joint effusion and popliteal cysts, although cyst size did not correlate with the presence of joint effusion. As reported earlier, Lyme arthritis can be associated with Baker cysts [14]. We found two Baker cysts in four patients with Lyme arthritis. Both clinicians and paediatric radiologists should be aware of this possible association, particularly in geographic regions where Lyme arthritis is endemic. 

Trauma history may not be a frequent cause of Baker cysts in children [4] but should nevertheless be considered, as one patient with degenerative meniscopathy from our study indicates. Some other rare causes of Baker cysts, such as pigmented villonodular synovitis [15], or some conditions mimicking popliteal cysts, including popliteal artery aneurysm or venous aneurysm, popliteal deep vein thrombosis, or soft-tissue masses [16–18], were not seen in our study group. 

The main focus of our study was on ultrasound imaging, as US is widely available, non-invasive, and effective in terms of diagnostic value and cost. An earlier study on adult patients reported 100% sensitivity of US for 21 cases of popliteal cysts, as compared with MRI, based on fluid detection between the semimembranosus and medial gastrocnemius tendons in communication with a posterior knee cyst [18]. The high rate of echogenic Baker cysts seen in our arthritis patients may help to guide diagnostic efforts in patients with popliteal cysts of unknown aetiology.

Although MRI was only performed in 20% of our study population, we discovered no significant discrepancy between the two modalities in detecting popliteal cysts. However, MRI can objectively demonstrate a wider range of intra- and extra-articular pathologies and should present the next diagnostic step when US fails to comprehensively answer the clinical question. 

In spite of long-standing, and continuing, controversies over the origin of synovial cysts, Baker cysts are thought to present protrusions of the gastrocnemius-semimembranous bursa and the subgastrocnemius bursa. The question of whether Baker cysts in children do or do not communicate with the joint space is a matter of controversial discussions. Widely disconcordant data are reported from different studies. In most cases, a slit-like fluid-signal extension can be demonstrated leading from the cyst towards the articular capsule and ending in the close vicinity of the latter. A wide-open channel connecting the cyst to the joint space is rarely seen even in adults. The retrospective mode of data analysis is a major limitation of our study in this respect. In our study group, the majority of Baker cysts were assessed as showing such communication on US, and more frequently on MRI. The inconsistency observed between the two modalities can most likely be attributed to the retrospective study design. Digitally archived MRI studies facilitate the retrospective review of the complete imaging study. On the other hand, selection and documentation of representative US scans relies heavily on the personal experience and the given circumstances at the time of the examination and are far less suitable for retrospective evaluation. Furthermore, probe palpitation of the Baker cyst for demonstrating communication with the internal joint space has so far not been part of routine US assessment at our institution, further limiting the chance of detecting cyst communication on ultrasonography. A definite answer to the question whether or not there is indeed a communication between the two compartments would probably require an arthrographic approach with cyst puncture. In our study, unlike previously reported data [4], the presence of joint effusion was significantly linked to the prevalence of a popliteal cyst, while there was no relation between the presence of effusion and cyst size. Here again the retrospective study mode poses a limitation, as data on quantification of joint effusion and its distribution within the knee joint compartments could not be reliably obtained with retrospective analysis from US reports and image documentation.

Fortunately, most Baker cysts in children regress spontaneously or after successful treatment of the underlying pathogenic cause [19]. For the time being, most ultrasonographers will feel assured to diagnose a popliteal cyst if they can demonstrate a cystic lesion in the medial popliteal fossa with the typical slit-like curved apex in close spatial relation to the posteromedial knee joint space.

## 5. Conclusion

Baker cysts, though generally rare in children, show a relatively high prevalence in certain paediatric subpopulations, namely, in patients with arthritis and benign joint hypermobility syndrome. Clinical examination can effectively limit the number of false-negative imaging studies performed for Baker cysts. The diagnosis of popliteal cysts can be established on ultrasound with high diagnostic accuracy, which generally is sufficient for the diagnostic workup of popliteal swelling. Cysts with echogenic internal signal are a frequent finding in arthritic knee joints and should trigger further diagnostic steps in children without known history of arthritis. Magnetic resonance imaging supports diagnosis in these patients and facilitates whole-organ assessment. On the basis of our data, most Baker cysts in paediatric patients apparently communicate with the knee joint space.

## Figures and Tables

**Table 1:** Between-groups comparison of ultrasonography and MRI findings in 80 patients with clinically suspected Baker cysts.

	Patients with arthritis *n* = 17	Patients with joint hypermobility *n* = 6	Others *n* = 57	*P*
Age, years	10.4 ± 4.4	6.6 ± 2.5	8.2 ± 4.9	.075
Female sex, *n* (%)	8 (47%)	0 (0%)	26 (46%)	.090
Ultrasonography				
Joints examined, *n*	28	12	107	
BC all, *n*	16	7	32	.007
BC bilateral, *n*	4	1	4	.099
Cyst volume, mL	3.9 ± 3.3	3.7 ± 2.0	3.2 ± 3.0	.471
Anechogenic cysts, *n* (% BC all)	6 (37%)	4 (57%)	26 (81%)	.009
Echogenic cysts w/o septations, *n* (% BC all)	10 (63%)	3 (43%)	6 (19%)	.009
Synovitis, *n* (% BC all)	8 (50%)	0 (0%)	0 (0%)	
BC and joint effusion (% BC all)	11 (69%)	1 (14%)	3 (9%)	<.001
BC communication with joint space, *n* (% BC all)	8 (50%)	6 (86%)	21 (66%)	.264
MR imaging				
Joints examined, *n*	6	2	16	
BC all, *n*	5	2	7	.134
Cyst volume, mL	5.0 ± 2.4	4.0 ± 2.8	3.3 ± 3.1	.389
BC and joint effusion (% BC all)	4 (80%)	0 (0%)	1 (14%)	.056
BC communication with joint space, *n* (% BC all)	5 (71%)	2 (100%)	7 (100%)	.615

US: ultrasonography, MR: magnetic resonance, and BC: Baker cyst. *P* values were calculated with Chi-Square test, Fisher's exact test, and Kruskal-Wallis test (age, cyst volume), as appropriate.
